# Lipids Metabolism and Cardiometabolic Diseases

**DOI:** 10.3390/ijms242417460

**Published:** 2023-12-14

**Authors:** Melania Gaggini, Cristina Vassalle

**Affiliations:** 1 Istituto di Fisiologia Clinica, Italian National Research Council, Via Moruzzi 1, I-56124 Pisa, Italy; melania.gaggini@ifc.cnr.it; 2 Fondazione CNR-Regione Toscana G. Monasterio, Via Moruzzi 1, I-56124 Pisa, Italy

Cardiometabolic diseases (CMD) remains the major cause of morbidity and mortality in Western countries, with a marked increased in the last years [1], as its component (e.g., coronary heart disease, stroke, type 2 diabetes, dyslipidemia, and hypertension) have common etiological underlying mechanisms, risk factors, and bidirectional links. In particular, lipids are the regulators of the biological processes that are associated with normal cell function, metabolism, and distribution; thus, changes in lipid components can have profound effects on cell function, the immune system, antioxidant defenses, and the inflammatory response [2]. For these reasons, the assessment of the traditional lipid profile, as well as lipid-lowering strategies, represented, for a long time, one of the main well-recognized pillars of cardiovascular prevention and treatment [2]. Nonetheless, all mechanisms which characterized different lipid species as inductors of, or protectors against, atherosclerosis and CMD are not completely cleared, a fact which may be also critical in developing new and more reliable therapeutic targeted strategies. Moreover, other alternative lipid species need to be evaluated as potential CMD biomarkers or therapeutic objectives [3]. The advent of new tools and information technologies have made it feasible to analyze entire lipid profiles (lipidomics) in biofluids and tissues [4,5]. The determination of individual lipid characteristics (composition and abundance) in biosamples could be a powerful tool that could be used to understand the mechanisms of lipid-based diseases. However, it remains poorly understood as to which lipids and species (i.e., among ceramides, phosphocholine, lysophosphatidylcholines) are the main culprits in the pathogenesis and development of CMD. 

The present Special Issue in *IJMS*, entitled “Lipids Metabolism and Cardiometabolic Diseases”, includes a total of nine contributions (seven original articles and two reviews) providing new information on different aspects of the relationship between lipids and CMD.

Djekic et al. evaluated the changes in the proportions of the HDL subclasses and pPON1 within HDL in patients with ST-segment elevation acute myocardial infarction (STEMI) against healthy controls [6]. Their results demonstrated that STEMI patients are characterized by the impaired maturation and functionality of HDL particles and alterations in the distribution of pPON1 within HDL, as well as an increased proportion of small HDL3b and HDL3c subclasses and higher pPON1 within HDL3, suggesting the association of these alterations in the lipid asset with the oxidative stress, high level of inflammation, and dyslipidemia observed in the STEMI patients.

Torres-Paz et al. investigated miR-221-5p, miR-21-5p, and miR-155-5p expression in monocytes and their role in coronary artery disease (CAD) [7]. The results showed that monocytes from CAD patients increased the expression of miR-21-5p and miR-221-5p and decreased the expression of miR-155-5p and NOS3; however, only the overexpression of miR-21-5p and miR-221-5p was found to be associated with an increased CAD risk. Interestingly, metformin downregulated the expression of miR-21-5p and miR-221-5p, suggesting a protective role of this drug in CAD through effects mediated by miRNA. Another miRNA, the miRNA-122, the most abundant liver-specific miRNA which protects against hepatic steatosis and inhibits cholesterol and fatty acid synthesis in NAFLD, was the focus of the study by Liu et al., whose results suggested that the serum levels of miRNA-122 gradually increased according to the severity of the NAFLD, providing a new explanation for the pathogenesis of the hepatic lipid deposition in NAFLD, characterized by an increased miRNA-122 extra-hepatocyte excretion and the enhancement of the stability of the miRNA-122 related to its binding to circPI4KB [8]. Always in the miRNA field, Castano et al. explore the potential for modulating obesity- and exercise-derived extracellular vesicles (EVs)-miRNA to treat the metabolic dysfunction associated with obesity in mice [9]. EV-miRNAs improve glucose intolerance and insulin resistance in obese mice similarly to high-intensity interval training stimulus, although only exercise improved cardiorespiratory fitness and decreased body weight; moreover, EV-miRNAs decreased fatty acid and cholesterol biosynthesis pathways in the liver, reducing hepatic steatosis and increasing insulin sensitivity, thus reducing glycemia and triglyceridemia. In view of these results, the authors suggest that manipulation of EV-miRNAs has an interesting future potential for metabolic dysfunction in the clinical setting, for example, with application in patients with obesity and/or diabetes who are unable to exercise.

Fatty acid amide hydrolase (FAAH) is responsible for the hydrolysis of several important endogenous fatty acid amides, including the endogenous cannabimimetic agent anandamide (AEA). In their study, Rajlic et al. evidenced that FAAH deficiency induces adverse cardiac effects (including increased inflammation, cardiomyocyte loss, and deteriorated left ventricular function) after myocardial ischemia and reperfusion in mice [10]. The authors also reported that as endocannabinoids (e.g., AEA) are also fatty acids and therefore affect receptors involved in fatty acid metabolism (such as PPAR-α), their increase may have negative effects on cardiomyocyte survival (due to PPAR-α activation); instead, restoration of the pathophysiological parameters in FAAH^−/−^ mice is obtained by blocking the AEA effect on PPAR-α. 

The study of Nieddu et al. applied targeted lipidomics to purified plasma lipoprotein fractions, showing the dysregulation of three lipid species (PE (38:6), SM(32:1), and SM (32:2)) between two groups of patients with “hard” or “soft” carotid plaques, homogenous in terms of lipid profile, glycemia, blood pressure, and pharmacological treatments [11]. Thus, these results evidence the utility of targeted lipidomics on purified lipoproteins to find new biomarkers of clinical relevance, which may help to further stratify patients according to acute clinical event risk. Always exploring the lipidomic field, Mocciaro et al. used an integrated “omics” approach (untargeted whole serum lipidomics, targeted proteomics, and lipoprotein lipidomics) to study lipoprotein remodeling and HDL composition in subjects with central obesity diagnosed with MetS (vs. controls), evidencing abnormalities of phospholipid metabolism in HDL, partially related to lecithin cholesterol acyltransferase, which appeared negatively associated with obesity and insulin resistance, thus adding further mechanistic insights concerning molecular aspects of the association between MetS and atherosclerosis [12]. 

The review by Gaggini et al. focusses on the non-traditional lipids (including non-HDL cholesterol, Castelli risk index 1 and 2, triglyceride-rich lipoprotein cholesterol, and atherogenic index of plasma), discussing the available knowledge on their potential as new additive biomarkers to better stratify CM risk in patients with hyperlipidemia, as well as their role as possible therapeutic targets in the clinical practice [13]. As CMD is a very complex condition, better knowledge of each of these indices based on the different characteristics of lipid components used for their calculation may help to further stratify specific groups of patients in order to refine and reduce the residual risk, connecting bench to bedside and vice versa.

The review by Ravnskov et al. deals with the role of coagulation factors as key determinants of premature cardiovascular disease in familial hypercholesterolemia, reporting available data suggesting that high LDL-C is not the main cause of premature cardiovascular disease among familial hypercholesterolemia patients, as well as in the general population [14]. Specifically, evidence suggested that only a few familial hypercholesterolemia patients die prematurely and that the cause in the majority of cases is related to various coagulopathies which they have inherited as well.

The editors want to thank all the authors who contributed to the present Special Issue with manuscripts covering different innovative aspects in the relationship between lipids and CMD. In particular, the results reported in this Special Issue may help to foster a better understanding of the underlying mechanisms of this relationship, identify new promising biomarkers, developing clinical innovations in order to better stratify patients, and use reliable tools fitted for definite patient subgroups which may benefit more from their use along the way to the development of a stratified medical practice designed to accommodate the patient’s specific needs. 
